# FAMULATUR PLUS – A successful model for improving students' physical examination skills?

**DOI:** 10.3205/zma001097

**Published:** 2017-05-15

**Authors:** Achim Jerg, Wolfgang Öchsner, Harald Traue, Lucia Jerg-Bretzke

**Affiliations:** 1 University Hospital Ulm, Department of Psychosomatic Medicine and Psychotherapy, Medical Psychology, Ulm, Germany; 2 University Hospital Ulm, Department for Cardiac Anesthesiology, Ulm, Germany; 3 University of Ulm, Office of the Dean of Medical Studies, Ulm, Germany

**Keywords:** clinical skills, medical education, physical examination, practical training

## Abstract

**Introduction/Project description: **Several studies have revealed insufficient physical examination skills among medical students, both with regard to the completeness of the physical examination and the accuracy of the techniques used. FAMULATUR PLUS was developed in response to these findings. As part of this practice-oriented instructional intervention, physical examination skills should be taught through examination seminars and problem-oriented learning approaches. In order to ensure practical relevance, all courses are integrated into a 30-day clinical traineeship in the surgery or internal medicine department of a hospital (FAMULATUR PLUS).

**Research question:** Does participation in the FAMULATUR PLUS project lead to a more optimistic self-assessment of examination skills and/or improved performance of the physical examination?

**Methodology: **A total of 49 medical students participated in the study. The inclusion criteria were as follows: enrollment in the clinical studies element of their degree program at the University of Ulm and completion of the university course in internal medicine examinations. Based on their personal preferences, students were assigned to either the intervention (surgery/internal medicine; n=24) or the control group (internal medicine; n=25). All students completed a self-assessment of their physical examination skills in the form of a questionnaire. However, practical examination skills were only assessed in the students in the intervention group. These students were asked to carry out a general physical examination of the simulation patient, which was recorded and evaluated in a standardized manner. In both instances, data collection was carried out prior to and after the intervention.

**Results: **The scores arising from the student self-assessment in the intervention (IG) and control groups (CG) improves significantly in the pre-post comparison, with average scores increasing from 3.83 (±0.72; IG) and 3.54 (±0.37; CG) to 1.92 (±0.65; IG) and 3.23 (±0.73; CG). The general physical examination, which was only assessed among the students in the intervention group, was performed more completely after the instructional intervention than prior to it.

**Discussion: **On the basis of the data collected, it can be deduced that the FAMULATUR PLUS course has a positive effect on the self-assessment of medical students with regard to their physical examination skills. The validity of this conclusion is limited by the small sample size. In addition, it remains unclear whether a more positive self-assessment correlates with an objective improvement in physical examination skills.

## Zusammenfassung

**Einleitung/Projektbeschreibung:** Etliche Studien belegen die unzureichenden Kenntnisse Medizinstudierender in der körperlichen Untersuchung. Dies betrifft sowohl die Vollständigkeit wie auch die Technik der körperlichen Untersuchung. Vor diesem Hintergrund wurde die FAMULATUR PLUS entwickelt. Im Rahmen dieser praxisorientierten Lehrintervention soll die körperliche Untersuchung durch Untersuchungskurse und problemorientiertes Lernen vermittelt werden. Zur Gewährleistung der Praxisnähe sind alle Lehrveranstaltungen in eine 30tägige Famulatur in der Chirurgie oder Inneren Medizin integriert (FAMULATUR PLUS).

**Fragestellung: **Führt die Teilnahme an der FAMULATUR PLUS zu einer optimistischeren Selbsteinschätzung der Untersuchungsfertigkeiten und/oder verbesserten Durchführung der körperlichen Untersuchung?

**Methodik: **An der Studie nahmen insgesamt 49 Medizinstudierende teil. Einschlusskriterien waren die Immatrikulation im klinischen Studienabschnitt an der Universität Ulm sowie die Absolvierung des universitären Untersuchungskurses der Inneren Medizin. Die Studierenden entschieden sich nach individueller Präferenz für die Zuordnung zur Interventions- (Chirurgie/Innere Medizin; n=24) oder Kontrollgruppe (Innere Medizin; n=25). Die Selbsteinschätzung hinsichtlich der Fertigkeiten in der körperlichen Untersuchung wurde mittels eines Fragebogens für alle Studierenden evaluiert. Im Gegensatz dazu erfolgte die Erhebung der praktischen Untersuchungsfertigkeiten nur bei den Studierenden der Interventionsgruppe. Diese wurden aufgefordert, eine allgemein-orientierende körperliche Untersuchung am Simulationspatienten durchzuführen, welche aufgezeichnet und standardisiert ausgewertet wurde. Beide Erhebungen erfolgten prä- und postinterventionell.

**Ergebnisse: **Die Selbsteinschätzung der Studierenden von Interventions- (IG) und Kontrollgruppe (KG) verbessert sich signifikant im Prä-Post-Vergleich von den Durchschnittsnoten 3,83 (±0,72; IG) und 3,54 (±0,37; KG) auf 1,92 (±0,65; IG) und 3,23 (±0,73; KG). Die nur bei den Studierenden der Interventionsgruppe erhobene allgemein-orientierende körperliche Untersuchung wird nach der Lehrintervention vollständiger durchgeführt als zuvor.

**Diskussion: **Aus den erhobenen Daten lässt sich ein positiver Effekt der FAMULATUR PLUS auf die Selbsteinschätzung Medizinstudierender hinsichtlich ihrer Fertigkeiten in der körperlichen Untersuchung ableiten. Limitiert wird die Aussagekraft durch die geringe Stichprobengröße. Zudem bleibt offen, ob eine positivere Selbstevaluation mit einer

## 1. Introduction

The assessment of a patient's medical history and the physical examination of a patient are essential medical skills, as evidenced by the fact that more than three-thirds of all diagnoses can be made on the basis of medical history and physical examination alone [31], [35]. The inadequacy of the physical examination training given to young physicians is at odds with its importance. This concerns both the completeness of the examination and the accuracy of the physical examination techniques used. Haring and colleagues have shown that medical students only use about 60 percent of the techniques expected when performing a general physical examination [17]. The same is true of physicians [37]. What is alarming about this is that essential examinations are often not performed at all (e.g., blood pressure measurement). Furthermore, medical professionals also exhibit a lack of skills when it comes to the examination techniques they use. This is well-documented with regard to auscultation of the heart. For example, only 37 percent of the physicians investigated detected a mitral stenosis [39]. Other studies have yielded similar results [8], [12], [13], [26], [41]. Although the publications cited originated abroad, similar results are to be expected among German medical students [25]. But what are the reasons for this lack of physical examination skills? Certainly, they are multifaceted and include organizational reasons such as shortened patient hospital stays or time and cost pressure in hospitals [7], [14], [16], [27], [36]. Physicians' faith in technology and a lack of supervision of medical students during physical examinations also favor undesirable developments [4], [43]. 

## 2. Project presentation

The FAMULATUR PLUS project was initiated in order to counteract the developments described above with regard to physical examinations on a small scale. The core objective of this project is to supplement a clinical traineeship (FAMULATUR) with lectures/seminars (PLUS). Thus, both clinical examination courses and problem-oriented learning (POL) seminars are offered as part of the FAMULATUR PLUS course. The distinguishing feature of this concept is the complete integration of all lectures/seminars into a 30-day clinical traineeship in the surgical or internal medicine department of a hospital. The close interlinking of teaching and inpatient care during the clinical traineeship serves to facilitate the transfer of knowledge from theory to practice. Each week of the clinical traineeship is clearly structured in advance and focuses on a particular part of the body (e.g., the abdomen). The week begins with a physical examination course under the direction of a physician. The objective of this course is to impart essential examination techniques based on the National Competence-Based Catalog of Learning Objectives (NKLM). The lecturers first demonstrate the examination technique to be learned before the students implement it in practice. The lecturer then provides the students with critical feedback and suggestions for improvement. In order to consolidate the knowledge conveyed in the examination courses, students are encouraged to perform independent examinations on patients over the course of the week. These examinations must also be documented in the respective patient's medical history and examination forms and signed off on by the ward physician. This allows knowledge deficits to be identified and corrected. The POL takes place at the end of each week of the clinical traineeship. Based on a clinical case study (e.g., a patient with acute abdominal pain), lists of possible differential diagnoses are compiled by the medical students. Subsequently, they arrive at a preliminary diagnosis by establishing a fictitious medical history and performing a physical examination. The POL serves to emphasize the need for establishing a thorough medical history and performing a physical examination during the diagnostic process. For a more detailed description of the FAMULATUR PLUS course, please refer to our previous publication [22].

## 3. Research question

The focus of this study is to determine whether the FAMULATUR PLUS course leads to more optimistic self-assessment by participating students with regard to their physical examination skills. We also want to test the hypothesis that participation in the instructional intervention results in improved physical examination performance. 

## 4. Methodology

### 4.1. Sample

During the study period from August 2014 to September 2015, 49 medical students were recruited through the University of Ulm's medical students' association and the project website. All study participants were enrolled in the clinical studies course at the University of Ulm and had successfully completed the university course in internal medicine examinations. Students were assigned to the intervention (n=24) and control groups (n=25) based on their personal preferences. The subjects in the control group participated in a 30-day clinical traineeship in an internal medicine department of a German hospital of their choosing, but did not participate in the instructional intervention. 

#### 4.2. Data collection

##### 4.2.1. Self-assessment questionnaire

**Figure 1:**
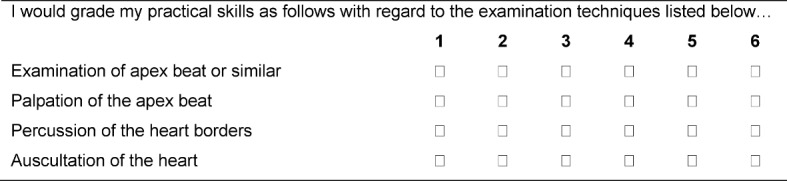
Excerpt from the self-assessment questionnaire based on the examination of the heart.

A questionnaire based on the work of Haring et al. was developed to evaluate the self-assessment of each participant [18]. This questionnaire was pretested on ten medical students and then finalized. The questionnaire consisted of three sections with a total of 58 items which had to be addressed by the students in both groups before and again after the intervention. While the first part of the questionnaire focused on general and demographic information, the second was used to evaluate individual examination techniques (inspection, palpation, percussion, and auscultation). Based on the organs and structures examined, the survey of the examination techniques was organized into the following categories: General and Vital Parameters, Head and Neck, Thorax, Heart, Circulation and Pulse, Abdomen and Groin, Extremities, and Neurology. The self-assessment was based on a six-point Likert scale in line with the German grading system, ranging from 'very good' (1) to 'fail' (6). See the illustration in Figure 1 (Fig. 1) for further details.

In the final section of the questionnaire, students were asked to make a global assessment of their physical examination skills. Here, too, the evaluation was based on the German grading system. The students in the intervention and control groups were asked to complete the questionnaire one day prior to and one day after completing the clinical traineeship. Only fully completed forms were included in the evaluation, which meant that all student questionnaires from the intervention group but only thirteen questionnaires from the control group could be included.

##### 4.2.2. General physical examination

In contrast to the questionnaire described above, data on the general physical examination were only collected from the students in the intervention group. These students were given 15 minutes to perform a physical examination of the simulation patient. In each case, the examination was recorded using a video camera and evaluated by a physician who was not involved in the project. The evaluation criteria in this regard included the completeness of the examination as well as the correct implementation of the examination techniques. The test was documented in anonymized format by means of an evaluation sheet, which comprised the following categories: "examination not performed," "examination performed correctly," and "examination performed incorrectly." Since the latter category was not used, it was not taken into account during the evaluation of the test. A point was assigned for each correctly performed examination and expressed as a score out of the maximum number of points, i.e. if all required examinations are performed correctly (see 5.2). The horizon of expectation was based on the works of Haring and colleagues [17], [18]. The items surveyed corresponded to those evaluated in the second section of the self-assessment questionnaire (see 4.2.1). Test design and evaluation were based on the literature [5], [11], [44].

##### 4.2.3. Statistical methods

The statistical evaluation of the collected data was carried out using the IBM SPSS Statistics Version 22 and Microsoft Office Excel 2007 data processing programs. The significance level was set at five percent (p≤0.05). Mann-Whitney and Wilcoxon tests for independent samples were used for the statistical comparison of the intervention group and control group self-assessments.

## 5. Results

### 5.1. Demographic data

A total of 49 medical students were included in the study and assigned to the intervention (n=24) or control group (n=25). The gender distribution, age structure, and academic experience of the students in both groups was homogenous. Participants were predominantly female, between 23 and 25 years old, and enrolled in the sixth semester at the University of Ulm.

#### 5.2. Self-assessment questionnaire

The students in the control and intervention groups were required to assess their physical examination skills prior to and after the clinical traineeship or instructional intervention by means of a specially developed questionnaire (see 4.2.1).

The evaluation of the questionnaire shows similar results for the data before and after the intervention. There is no significant difference between the pre-intervention self-assessment of the intervention group students and that of the control group students (see Table 1 (Tab. 1)).

However, the results are different for both groups with regard to the post-intervention analysis. Students in the intervention group are more optimistic with regard to their examination skills across all categories than prior to completing the FAMULATUR PLUS course, with the average total score improving to 1.92 (± 0.65), for example. The participants of the control group also perceived their skills as better after the clinical traineeship than before. However, the differences between the average scores in the control group are less pronounced in the pre-post comparison than those in the intervention group. In addition, the examination of the heart is assessed more pessimistically in this group after the clinical traineeship than prior to it. The overall post-intervention score of the students in the control group is 3.23 (± 0.73). In conclusion, however, it should be noted that only the post-intervention data on the examination of the heart, abdomen and groin, those pertaining to the examination of the nervous system, and the overall score are significant (p≤0.05). Accordingly, the self-assessment of the students in the intervention group can only be said with absolute certainty to be more positive than that of the students in the control group in these categories. 

A detailed comparison of the values and their significance can be found in Table 1 (Tab. 1).

#### 5.3. General physical examination

In order to assess their practical physical examination skills, students in the intervention group were asked to perform a full-body examination on a simulation patient. The examinations were recorded and evaluated by an independent physician in a standardized manner. Only those examinations that were performed correctly were included in the evaluation (see 4.2.2). 

The pre-post comparison shows that the physical examination is more complete after the instructional intervention than before. The discrepancy between data collected prior to and after the FAMULATUR PLUS course is particularly marked for the examinations of head and neck (+59%), heart (+49%), thorax (+45%), and abdomen (+43%). While, for example, only one of the six required head and neck examinations was performed prior to the instructional intervention, five of them were completed afterwards. The increases in the number of examinations of the circulatory and neurological systems performed (+32% each) as well as those of the vital parameters (+24%) and extremities (+23%, not significant) are less pronounced (see Table 2 (Tab. 2)).

## 6. Discussion

The declared goal of the FAMULATUR PLUS project is to address the deficits in the physical examination skills of medical students described above. This study investigated whether the developed instructional intervention is an effective means of improving self-assessment and practical physical examination skills.

The evaluation of the self-assessment confirms the expectation that the self-assessment of the students in the intervention group improves in the pre-post comparison. The fact that this improved self-assessment is owed to the instructional intervention is highlighted by the comparison with the subjects of the control group, where no comparable effects were observed. Apart from this, it seems plausible for practical training - as offered by the FAMULATUR PLUS course - to result in improved self-assessment. This general assumption is also confirmed in the literature [29], [30]. There are also a number of other studies that emphasize the positive effects of practical training programs on self-assessment [1], [14], [15], [19], [28], [38], [42]. Moreover, FAMULATUR PLUS participants must regularly perform physical examinations on patients. The documentation of these examinations in the form of completed medical history and examination forms must be signed off on by the ward physician. This ensures that examination skills are supervised on practiced on a regular basis. Both result in optimized self-assessment [6], [9], [20], [21], [40]. However, one criticism of this approach is that access to self-assessment may be subject to cognitive limitations [34], [40]. Furthermore, self-assessment and the quality of the physical examination are not correlated [2]. 

Therefore, the subjective self-assessment should be supplemented by a more objective parameter, namely the general physical examination. The analysis of the physical examination shows a significantly improved examination performance among students who participated in the instructional intervention. This improvement is reflected both in a more complete and a more technically correct implementation of the examination techniques. This is primarily due to the practice-oriented training (e.g., examination courses) offered. It is known that students who have completed a training use a greater number of examination techniques than those who have not completed a training [3], [32], [33]. The same also applies to their performance in practical tests [23]. In addition, the very act of participating in the study is likely to have a positive effect [24]. Notwithstanding this, one shortcoming of the pilot study is the lack of a control group. However, for organizational reasons, it was not possible to get the students in the control group to perform examinations, especially since the response rate for the self-assessment questionnaires failed to meet expectations.

The overall validity of the study is limited by the small sample size and possible positive selection bias for particularly motivated students with regard to participation in the instructional intervention. In addition, no information was collected on previous experience in the form of further clinical traineeships. However, it cannot be assumed that the clinical traineeship is the only factor responsible for the improvement in examination skills [14], [33], [43]. Details about the hospitals in which the students in the control group completed their clinical traineeships were also not evaluated. For example, it is conceivable that a clinical traineeship in a university hospital might include more practical teaching (e.g., bedside teaching) than one completed in a smaller, regional hospital. The pre-post design of the study must also be questioned since it is susceptible to response-shift biases [10]. Last but not least, a more detailed evaluation of the contribution of POL to long-term learning success is also needed. 

## 7. Conclusion and outlook

The pilot study shows that the FAMULATUR PLUS course is an effective means of improving the self-assessment of medical students with regard to their physical examination skills. However, further studies are needed for more detailed verification. For example, the results of the general physical examination must be checked using control groups. 

## Ethics

The research project was approved by the Ethics Committee of the University of Ulm.

## Funding

The study was supported by the Faculty of Medicine of the University of Ulm as part of its teaching research funding.

## Competing interests

The authors declare that they have no competing interests.

## Figures and Tables

**Table 1:**
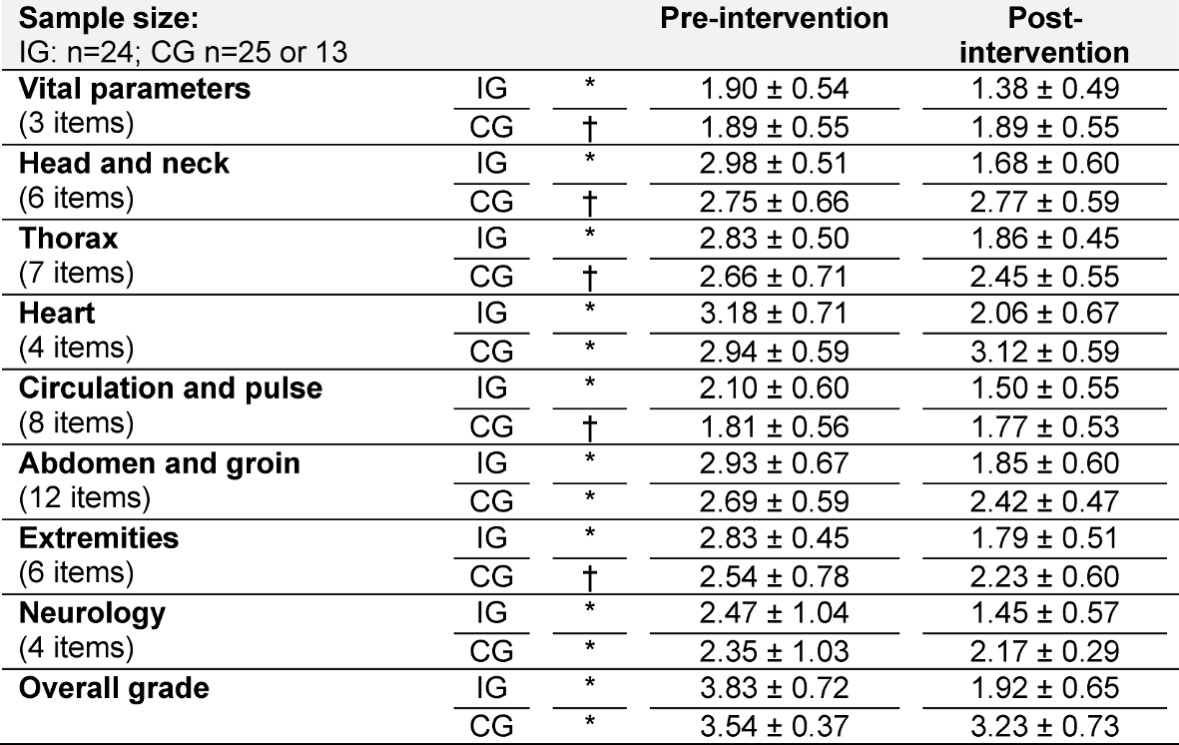
Self-assessment of examination skills using a six-point Likert scale in line with the German grading system (1: very good; 6: fail). The table shows the average score (±standard deviation; *significant or † not significant in the pre-post comparison) of the students in the intervention (IG; n=24) and control groups (CG; n=25; analyzable: 13).

**Table 2:**
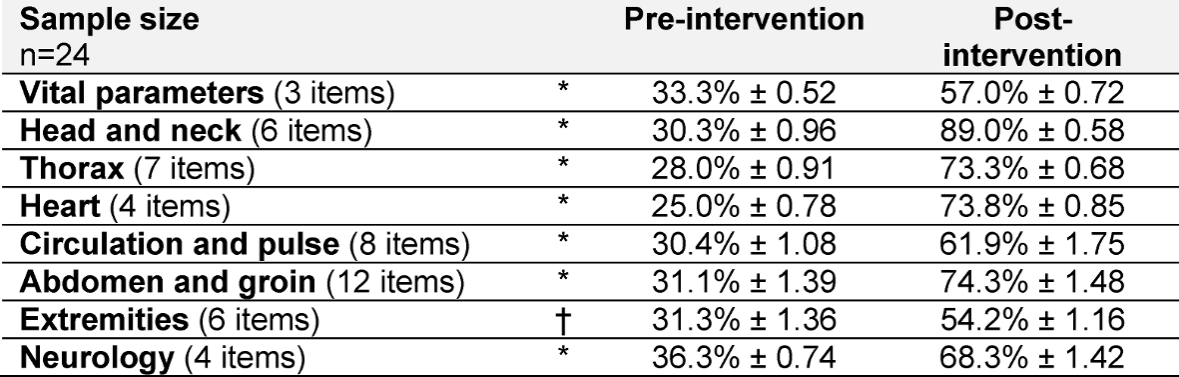
General physical examination. Students (n=24) were asked to perform a full-body examination on a simulation patient while being recorded on video. The examination videos were evaluated using a standardized evaluation sheet by a physician who was not involved in the project. The table shows the number of correctly performed examinations in relation to the maximum value (mean±standard deviation; *significant or †not significant in the pre-post comparison).
